# Mice Deficient in Transmembrane Prostatic Acid Phosphatase Display Increased GABAergic Transmission and Neurological Alterations

**DOI:** 10.1371/journal.pone.0097851

**Published:** 2014-05-20

**Authors:** Heidi O. Nousiainen, Ileana B. Quintero, Timo T. Myöhänen, Vootele Voikar, Jelena Mijatovic, Mikael Segerstråle, Annakaisa M. Herrala, Natalia Kulesskaya, Anitta E. Pulkka, Tanja Kivinummi, Usama Abo-Ramadan, Tomi Taira, T. Petteri Piepponen, Heikki Rauvala, Pirkko Vihko

**Affiliations:** 1 Department of Clinical Chemistry, University of Helsinki and Helsinki University Hospital Laboratory, Helsinki, Finland; 2 Division of Pharmacology and Toxicology, Faculty of Pharmacy, University of Helsinki, Helsinki, Finland; 3 Neuroscience Center, University of Helsinki, Helsinki, Finland; 4 Department of Biosciences, Faculty of Biological and Environmental Sciences, University of Helsinki, Helsinki, Finland; 5 Experimental MRI Laboratory, Department of Neurology, Helsinki University Central Hospital, Helsinki, Finland; 6 Institute of Biomedicine, University of Helsinki, Helsinki, Finland; 7 Department of Veterinary Biosciences, Faculty of Veterinary Medicine, University of Helsinki, Helsinki, Finland; University of Wuerzburg, Germany

## Abstract

Prostatic acid phosphatase (PAP), the first diagnostic marker and present therapeutic target for prostate cancer, modulates nociception at the dorsal root ganglia (DRG), but its function in the central nervous system has remained unknown. We studied expression and function of TMPAP (the transmembrane isoform of PAP) in the brain by utilizing mice deficient in TMPAP (PAP*^−/−^* mice). Here we report that TMPAP is expressed in a subpopulation of cerebral GABAergic neurons, and mice deficient in TMPAP show multiple behavioral and neurochemical features linked to hyperdopaminergic dysregulation and altered GABAergic transmission. In addition to increased anxiety, disturbed prepulse inhibition, increased synthesis of striatal dopamine, and augmented response to amphetamine, PAP-deficient mice have enlarged lateral ventricles, reduced diazepam-induced loss of righting reflex, and increased GABAergic tone in the hippocampus. TMPAP in the mouse brain is localized presynaptically, and colocalized with SNARE-associated protein snapin, a protein involved in synaptic vesicle docking and fusion, and PAP-deficient mice display altered subcellular distribution of snapin. We have previously shown TMPAP to reside in prostatic exosomes and we propose that TMPAP is involved in the control of GABAergic tone in the brain also through exocytosis, and that PAP deficiency produces a distinct neurological phenotype.

## Introduction

There are two isoforms of prostatic acid phosphatase enzyme: secretory (sPAP) and transmembrane (TMPAP) [1], [2] splice variants encoded by the same gene (*ACPP*). TMPAP is a type 1 transmembrane protein with 5′ectonucleotidase activity and is also widely expressed in non-prostatic tissues in both sexes. TMPAP contains an N-terminal phosphatase activity domain which is extracellular when TMPAP is in the plasma membrane and intra-luminal when it is trafficking in vesicles, and a C-terminal domain with a cytosolic tyrosine-based endosomal-lysosomal (including MVE) targeting signal motif (YxxΦ). TMPAP also colocalizes with exosomal markers flotillin and Lamp-1 [1], is released in exosomes and interacts in prostate cells with the SNARE-associated protein snapin [3]. Snapin interacts biochemically with t-snare SNAP-25, which belongs to the presynaptic release machinery [4], and is critical for presynaptic homeostatic plasticity [5]. Additionally, SNAP-25 is involved in slow clathrin-dependent endocytosis [6], and alterations in clathrin-mediated endocytosis and clathrin-dependent membrane and protein trafficking have been hypothesized as core pathophysiological mechanisms in neuropsychiatric disorders such as schizophrenia and bipolar disorders [7]. When exosomes were described for the first time, it was shown that exosomes display 5′-ectonucleotidase activity [8].

In the DRG, TMPAP functions as a 5′-ectonucleotidase and produces adenosine [2], [9] that suppresses pain by activating adenosine A_1_-receptors. An intraspinal injection of sPAP has efficient and long-lasting effects against pain sensation in healthy animals, as well as pain reliving effects in animals sensitized by nerve injury [9]. PAP*^−/−^* mice have increased sensitivity for the development of chronic inflammatory and neuropathic pain [9], [10]. Given the endosomal/lysosomal and exosomal localization of TMPAP along with its known role in pain regulation in the peripheral nervous system, this observation prompted us to characterize PAP expression and function in the central nervous system in more detail. We conclude that through an alteration in the mechanisms involving vesicular traffic, especially exocytosis, the lack of PAP produces distinct endophenotypes, such as altered prepulse inhibition, that are also seen in animal models of several mental disorders.

## Materials and Methods

### Ethics Statement

All procedures and Experiments involving mice were approved by ELLA - The National Animal Experiment Board of Finland. The project license numbers are STH705A/ESLH-2009-08353/Ym-23 and 044/11.

### PAP Deficient Mice

PAP*^−/−^* mice were generated by removing exon 3 (*PAP^Δ3/Δ3^)* of the prostatic acid phosphatase gene (*PAP, Acpp*), completely abolishing expression of sPAP and TMPAP proteins encoded by the *Acpp* gene, respectively [10]. PAP*^−/−^* mice have been backcrossed to C57BL/6J strain (Harlan Laboratories, Inc.) for 16 generations. PAP*^−/−^* male mice were analyzed with age-matched C57BL/6J wild-type (WT) male mice as controls.

### Magnetic Resonance Imaging

Mice 12-month-old (WT (n = 5) and PAP*^−/−^* (n = 5)) and 2-month-old (WT (n = 4) and PAP*^−/−^* (n = 4)) were anesthetized with isoflurane for the imaging experiment. MRI studies were performed with a 4.7 T scanner (PharmaScan, Bruker BioSpin, Ettlingen, Germany) using a 90-mm shielded gradient capable of producing a maximum gradient amplitude of 300 mT/m with an 80-µs rise time. A linear birdcage radio frequency coil with an inner diameter of 19 mm was used. After shimming and scout images, coronal T2-weighted 2D images encompassing the whole brain were acquired with using the standard Bruker technique of fast spin echo sequence; rapid acquisition with relaxation enhancement (RARE) sequence (TR/TEeff, 3800/80 milliseconds; Rare factor 8, matrix size, 256×256; field of view, 23×23 mm2; 15 slices, slice thickness 0.5 mm). The body temperatures of the animals were maintained by using a MRI-compatible heating pad (Gaymar Industries,Orchard Park, NY, USA). Lateral ventricle images were processed using the manual tracing tool provided by ParaVision 4.0 (Bruker BioSpin, Ettlingen, Germany). Manually delineated regions of interest for the right and the left lateral ventricle in each slice were summed up and multiplied by slice thickness yielding the right and left lateral ventricle volumes. To calculate the total brain volume, the coronal sections obtained by MRI were analyzed using ImageJ 1.48f program (Wayne Rasband, National Institutes of Health, USA). Analysis of the MRI images was performed so that the person analyzing the images did not know the genotypes of the animals. The area corresponding to the brain was selected using the free hand selection tool, and a mask was generated (excluding cerebellum and olfactory bulbs). Image calculation was performed for each slice of the image stack, to the resulting images the automatic threshold adjustment was applied, noise was removed and holes were filled. The area for each image slice was calculated using the analyze particle tool. Once all brain image slices were measured, the total brain volume was calculated as the sum of every area multiplied by the slice thickness. Groups were divided by genotype and age, and Student’s t test was performed to compare brain size.

### Behavioral Tests

#### Mice in behavioral tests

All mice (PAP^−/−^ and WT) assayed in behavioral tests were 2.5 to 3.5-month-old.

#### Video tracking

The mice were video-tracked by Noldus EthoVision XT 8.0 system (Noldus Information Technology, Wageningen, The Netherlands) during the elevated plus-maze, Y-maze, water maze, forced swim and tail suspension tests. The distance travelled by the subjects and the time spent in pre-defined zones were recorded.

#### Elevated plus maze

Elevated plus maze test (EPM) was used to measure unconditioned anxiety-like behaviour in mice (PAP^−/−^ n = 22, WT n = 23). The test was conducted as described in [11]. Briefly, the maze consisted of two open arms (30×5 cm) and two enclosed arms (30×5 cm, inner diameter) connected by central platform (5×5 cm). The maze was raised to 40 cm above the floor. The floor of each arm was light grey and the closed arms had transparent (15 cm high) side- and end-walls. The illumination level in all arms was ∼150 lx. The mouse was placed in the center of the maze facing one of the enclosed arms and observed for 5 minutes. The latency to the first open arm entry, number of open and closed arm entries (four paw criterion) and the time spent in different zones of the maze were measured.

#### Prepulse inhibition of acoustic startle response

Sensorimotor gating (PPI) was measured in commercially available system (Med Associates, St. Albans, GA, USA). For initial screening 9 PAP^−/−^ and 9 WT mice were tested. The method is described in details in [11]. Mice were enclosed in a transparent plastic tube (Ø 4.5 cm, length 8 cm) that was placed in the startle chamber (Med Associates) with a background white noise of 65 dB and left undisturbed for 5 minutes. Testing was performed in 12 blocks of 5 trials and five trial types were applied. One trial type was a 40-ms, 120-dB white noise acoustic startle stimulus (SS) presented alone. In the remaining four trial types the startle stimulus was preceded by the acoustic prepulse stimulus (PPS). The 20-ms PPS were white noise bursts of 68, 72, 76 and 80 dB. The delay between onset of PPS and SS was 100 ms. The 1^st^ and 12^th^ block consisted of SS-alone trials. In remaining blocks the SS and PPS+SS trials were presented in pseudorandomized order such that each trial type was presented once within a block of 5 trials. The inter-trial interval ranged between 10 and 20 seconds. The startle response was recorded for 65 ms starting with the onset of the startle stimulus. The maximum startle amplitude recorded during the 65-ms sampling window was used as the dependent variable. The startle response was averaged over 10 trials from blocks 2–11 for each trial type. The prepulse inhibition for each PPS was calculated by using the following formula: 100−[(startle response on PPS+SS trials/startle response on SS trials) ×100]. Finally, the average inhibition of the startle over all pre-pulse levels was used for analysis. Haloperidol was dissolved in saline and administered at the dose of 0.2 mg/kg i.p. 20 min before start of experiment. Number of mice used: PAP^−/−^ n = 15 (saline) +15 (haloperidol), WT n = 15 (saline) +14 (haloperidol).

#### Amphetamine- and MK-801-induced locomotor activity

The mice were adapted for 30 min in the open field arena (30×30 cm, Med Associates, St. Albans, VT, USA). After adaptation, the animals received an i.p. injection of D-amphetamine (Faculty of Pharmacy, University of Helsinki, Finland; 3 mg/kg; PAP^−/−^ n = 15, WT n = 17) or MK-801 (Research Biochemicals, Natick, MA, USA; 0.2 mg/kg; PAP^−/−^ n = 10, WT n = 10) and the activity (distance travelled) was monitored for 90 min.

#### Diazepam-induced loss of righting reflex

The mice (PAP^−/−^ n = 10, WT n = 10) were injected i.p. with high dose of diazepam (Orion Pharma, Espoo, Finland; 35 mg/kg) and checked for appearance of the loss of righting reflex by placing on their backs in a v-shaped trough as described previously [12].

#### Light-dark exploration

The test was carried out in the open field arena (30×30 cm, Med Associates, St. Albans, VT) equipped with infrared light sensors detecting horizontal and vertical activity (PAP^−/−^ n = 22, WT n = 23). The dark insert (non-transparent for visible light) was used to divide the arena into two halves, an opening (a door with a width of 5.5 cm and height of 7 cm) in the wall of the insert allowed animal’s free movement from one compartment to another. The light half was illuminated by two 40 W light bulb 50 cm above the floor (illumination in the center of the light compartment ∼1000 lx). Animal was placed in the light compartment and allowed to explore the arena for 10 minutes. Distance travelled, number of rearings, and time spent in different compartments were recorded.

#### Novelty-induced activity (Open Field)

The mice were released in the corner of novel open field arena (30×30 cm, Med Associates). Horizontal and vertical activity was recorded for one hour (light intensity ∼150 lx). Peripheral zone was defined as a 6 cm wide corridor along the wall (PAP^−/−^ n = 22, WT n = 23).

#### Y-maze

Spontaneous alternation performance was assessed in a symmetrical Y-maze under reduced light conditions (∼100 lx) (PAP^−/−^ n = 10, WT n = 10). Each arm was 30 cm long and 7 cm wide with transparent walls (15 cm high). Mice were allowed to explore the maze for 5 minutes. The number and the sequence of the arm entries were recorded. The measured variables were activity, defined as the number of arms entered, and percent alternation, calculated as the number of alternations (entries into three different arms consecutively) divided by the total possible alternations (i.e., the number of arms entered minus 2) and multiplied by 100.

#### Rotarod

The accelerating rotarod (Ugo Basile, Comerio, Italy) test was performed on two consecutive days (PAP^−/−^ n = 22, WT n = 23). The mice were given three trials a day with an inter-trial interval of 1 hour. Acceleration speed from 4 to 40 rpm over a 5-min period was chosen. The latency to fall off was recorded with the cut-off time set at 6 min.

#### Beam walking

The mouse is placed perpendicularly in the center of a horizontal round beam (covered with laboratory tape, outer diameter 2 cm, length 120 cm, divided into 12 sections and raised to 50 cm above the floor level). If the mouse is falling off in less than 10 sec, a new trial is started (max. 3 times). The retention time and the number of sections crossed on the beam during 2 min are measured (PAP^−/−^ n = 22, WT n = 23).

#### Forced swim test

The mouse was placed for 6 minutes in the glass cylinder (Ø 18 cm, height 25 cm) filled with water at 23±1°C to the height of 15 cm. The time of immobility (passive floating, when the animal was motionless or doing only slight movements with tail or one hind limb, whereas the animal was judged to be active when struggling, climbing or swimming using all four paws) was measured in 2 min bins.

#### Tail suspension test

The mouse is fixed hanging by tail on the horizontal bar with adhesive tape. The time of immobility (no struggling) is measured during 6 min in 2 min bins (PAP^−/−^ n = 14, WT n = 12).

#### Fear conditioning

The experiments were carried out employing a computer-controlled fear conditioning system (TSE) (PAP^−/−^ n = 22, WT n = 23). Training was performed in a transparent acrylic cage (23 × 23 × 35 cm) within a constantly illuminated (∼ 100 lx) fear conditioning box. A loudspeaker provided a constant, white background noise (68 dB) for 120 s followed by 10 kHz tone (CS, 76 dB, pulsed 5 Hz) for 30 s. The tone was terminated by a footshock (US, 0.6 mA, 2 s, constant current) delivered through a stainless steel floor grid (Ø 4 mm, distance 10 mm). Two CS-US pairings were separated by a 30 s pause. Contextual memory was tested 24 h after the training. The animals were returned to the conditioning box and total time of freezing (defined as an absence of any movements for more than 3 s) was measured by infrared light barriers scanned continuously with a frequency of 10 Hz. The CS was not used during this time. Memory for the CS (tone) was tested 2 h later in a novel context. The new context was a similarly sized acrylic box with black non-transparent walls and smooth floor. A layer of wood chips under the floor provided a novel odour to the chamber. After 120 s of free exploration in a novel context the CS was applied for additional 120 s and freezing was measured as above.

#### Tube test

Tube test is commonly used to measure social dominance in mice (PAP^−/−^ n = 10, WT n = 10). Two unfamiliar mice of the same sex but different genotypes were placed in the opposite ends of a 30×3.8 cm (inner diameter) transparent plastic tube and released simultaneously. The match ended when one mouse completely retreated from the tube. The mouse remaining in tube is designated the winner, and the retreated mouse is the loser. Each animal was tested against all animals from the opposed group. The percent of retreated matches as well as aggressive postures were scored for each animal. Matches lasting more than 2 min or in which animals crossed over each other were not scored.

#### Resident-intruder test

Resident-intruder test was used to measure social interaction (PAP^−/−^ n = 10, WT n = 10). An intruder mouse (unfamiliar sex- and age-matched animal of C57BL/6JOlaHsd strain) was put in the cage where the test mouse had been acclimatizing for 30 min. Time spent in social activity (sniffing, following, hetero-grooming) and non-social activity (digging, self-grooming, and rearing) was recorded during 5 min observation.

#### Water maze

The system consisted of a black circular swimming pool (Ø 120 cm) and an escape platform (Ø 10 cm) submerged 0.5 cm under the water surface in the centre of one of four imaginary quadrants. The animals were released to swim in random positions facing the wall and the time to reach the escape platform (maximum time 60 s) and the swimming distance were measured in every trial. In addition, thigmotaxis, the time spent swimming within the outermost ring of the pool (10 cm from the wall) was measured. Two training blocks consisting of three trials each were conducted daily. The interval between trials was 4–5 min and between training blocks about 5 hours. The hidden platform remained in a constant location for 3 days (6 initial training sessions) and was thereafter moved to the opposite quadrant for 2 days (4 reverse training sessions). The probe trials were conducted approximately 18 h after the last initial and reverse training sessions. The mice were allowed to swim in the maze for 60 seconds without the platform available. Spatial memory in the probe trials was estimated by preference of swimming in the trained region (imaginary circular area of Ø 30 cm, around the previous platform location) over swimming in corresponding regions in the three other quadrants. After the 2^nd^ probe trial, the mice were tested for one block of 3 trials with the platform made visible in the quadrant not employed previously (PAP^−/−^ n = 4, WT n = 9).

### Statistics

The behavioural data were analysed using a factorial ANOVA design with genotype and treatment as between-subject factors. A repeated measures ANOVA was applied for analysis of activity data. Post-hoc analysis after significant ANOVA was carried out by means of Newman-Keuls test. Mann-Whitney U-test was used for analysis of non-normally distributed data (diazepam-induced LORR). For other experiments, data was analysed with either two-tailed t-test or with repeated measures ANOVA.

### Brain Dissection

The mice were sacrificed by decapitation and their brains placed on an ice-cooled brain matrix (Stoelting, Wood Dale, Illinois, USA). Two coronal cuts were made by razor blades at about 1.5 and −0.3 mm from the bregma according to the mouse brain atlas of Franklin and Paxinos [13]. From the obtained section the dorsal striatum was punched below the corpus callosum by using a sample corer (inner diameter of 2 mm). Dissected tissue pieces were immediately placed into frozen microcentrifuge tubes and after weighing they were stored at −80°C until assayed.

### Estimation of Monoamines and their Metabolites

NSD-1015 (BioChemika, Sigma, China) was dissolved in saline (0.9% NaCl solution) and given half an hour before the mice were decapitated. Concentrations of dopamine (DA), L-dihydroxyphenylacetic acid (DOPAC) and homovanillic acid (HVA) from brain samples were analyzed using HPLC with electrochemical detection as described by [14]. The values of monoamines and their metabolites are presented as nanograms per gram (ng/g) wet weight of tissue.

### In vivo Microdialysis

Guide cannulae (AgnTho’s, Lidingö, Sweden) were implanted stereotaxically (coordinates A/P = +0.6, L/M = +1.8 and D/V = −2.3; [13] under isoflurane anesthesia [15]. After surgery, mice were placed into individual test cages and allowed to recover in the cages for at least 5 days before the experiment. On the experiment day, a microdialysis probe (AgnTho’s, Lidingö, Sweden, 1 mm membrane, outer diameter 0.24 mm, 6 kDa cut-off, MAB 4.9.1 Cu) was inserted into the guide cannula, and the probe was perfused with Ringer solution (147 mM NaCl, 1.2 mM CaCl2, 2.7 mM KCl, 1.0 mM MgCl2, and 0.04 mM ascorbic acid) at the flow rate of 2 µl/min. After a 3-h stabilization period, the collection of microdialysis samples was started. The concentrations of DA, DOPAC and HVA were determined by HPLC with an electrochemical detector [15]. The column (Kinetex 2.6 µ, 4.6×50 mm C-18; Phenomenex, Torrance, CA, USA) was kept at 40°C, with a column heater (Croco-Cil; Bordeaux, France). The mobile phase consisted of 0.1 M NaH_2_PO_4_ buffer, pH 4.0, 100 mg/ml octanesulphonic acid, 8% methanol and 1.0 mM EDTA. The flow rate was 1.0 ml/min. Thirty five microliters of the dialysate sample was injected into the column with a Shimadzu SIL-20AC autoinjector (Shimadzu, Kyoto, Japan). Basal extracellular concentrations of neurotransmitters (not corrected with *in vitro* -recovery) were determined as mean of concentrations of three stable pre-drug samples collected during the first 60 minutes of sampling (variation <20%). Potassium stimulation was performed by manually switching the perfusion medium, Ringer, to the one containing 100 mM KCl, 27.5 mM NaCl, 1.2 mM CaCl2, 1.0 mM MgCl2, and 0.04 mM ascorbic acid. Haloperidol (0.2 mg/kg i.p.), D-Amphetamine (100 µM; Faculty of Pharmacy, University of Helsinki, Finland) and adenosine A_1_-antagonist 8-CPT (300 µM; Research Biochemicals, Natick, MA, USA) were given via dialysis fluid for 20 and 140 min., respectively. Adenosine A_1_-agonist GR79236X (1 mg/kg) was given ip.

### Sample Preparation for Immunohistochemistry

Mice were anesthetized using pentobarbital (100 mg/kg, Mebunat Vet, Orion Pharma, Espoo, Finland) and perfused using phosphate buffered saline (PBS) followed by a perfusion with 4% paraformaldehyde in 0.1 M phosphate buffer (PB), pH 7.4. After perfusion, the mice were decapitated and brains were removed, post-fixed with 4% paraformaldehyde and sucrose, and stored in 0.05% Na-azide in PBS at +4°C until sectioning into 40 µm free-floating cryosections with a microtome (Leica SM2010, Leica Microsystems Inc., Bannockburn, IL, USA).

### PAP Immunohistochemistry (IHC)

PAP IHC for the mouse brain was performed by using free-floating sections and modifying the protocol described in [16]. Shortly, the endogenous peroxidase activity was inactivated with 10% methanol and 3% H_2_O_2_ in PBS (pH 7.4) for 10 min, and non-specific binding was blocked with 10% normal horse serum (Product# S-2000, Vector laboratories, Burlingame, CA, USA) in PBS. The sections were incubated overnight at room temperature with Goat anti-ACPP antibody (dilution 1∶500 in 1% normal serum; Product # EB09390, Everest Biotech, Oxfordshire, UK), followed by washes with PBS. The slides were then incubated with horse anti-goat biotin conjugated secondary antibody (dilution 1∶500 in 1% normal serum; Product #BA9500, Vector laboratories) for 2 h in room temperature, followed by PBS washes and ABC incubation (Vectastain Elite ABC Kit, Product PK-6100, Vector laboratories). The brown color was developed with 0.05% 3,3′-diaminobenzidine and 0.03% H_2_O_2_ in PBS. Finally, the sections were transferred to objective glasses, dehydrated in alcohol series and mounted with Depex (BDH, Poole, UK). PAP*^−/−^* mouse served as a control for immunostainings. Immunohistochemistry photomicrographs were captured by a digital camera connected to the Olympus BX40 microscope and DP50 Digital Camera (Olympus Corporation, Tokyo, Japan) and corrections to brightness and contrast were made with Adobe Photoshop CS2 software (version 9.0, Adobe Systems Incorporated, Mountain View, CA, USA).

### Double-label Immunofluorescence

To detect the colocalization of PAP with GABAergic neurons, double-label immunofluorescence was used as described in [17]. The sections were washed with PBS, non-specific binding was blocked with 10% normal horse serum (Vector laboratories), and the sections were incubated overnight in Goat anti-ACPP antibody (dilution 1∶500 in 1% normal serum; Everest Biotech, Oxfordshire, UK). The slides were then incubated with donkey anti-goat Alexa Fluor 488 conjugated secondary antibody (dilution 1∶500 in 1% normal serum; Product #A11055, Invitrogen, Eugene, OR, USA) for 2 h in room temperature, followed by PBS washes. Then, the sections were incubated for 30 min in Goat normal serum (Product #S1000, Vector laboratories) followed by an overnight incubation with either rabbit anti-GAD 65/67 antibody (dilution 1∶500 in 1% normal serum; Product #G5163, Sigma-Aldrich), Rabbit anti-snapin (dilution 1∶500 in 1% normal serum, cat. no. 148 002, Synaptic Systems, Goettingen, Germany), Rabbit anti-synaptophysin (dilution 1∶500 in 1% normal serum,; Product # ab14692 Abcam, Cambridge, UK), or rabbit anti-CHMP2B (multivesicular bodies; dilution 1∶500 in 1% normal serum; Product #ab33174, Abcam, Cambridge, UK). After PBS washes, the slides were incubated with goat anti-rabbit Texas Red conjugated secondary antibody (dilution 1∶500 in 1% normal serum; Product #31506, Thermo Scientific, Rockford, IL, USA) for 2 h in room temperature. The slides were then transferred to objective glass, mounted with Vectashield with DAPI (Product #H-1000, Vector laboratories) and coated with coverslip. The sections were photographed using Leica TCS SP2 AOBS (Leica Microsystems GmbH, Wetzlar, Germany) equipped with an argon-He/Ne laser mounted on an inverted Leica DM IRE2 microscope (Leica Microsystems GmbH). Merging of images and minor corrections to brightness and contrast were made with Adobe Photoshop CS2 software (version 9.0, Adobe Systems Incorporated, Mountain View, CA, USA).

### Cloning of TMPAP from Striatal Neurons

Total RNA was isolated from Mouse Brain Striatum Neuronal Cells (Lonza, Basel, Switzerland) using TriReagent (Molecular Research Center, Cincinnati, OH, USA). RNA was reverse transcribed into cDNA and subsequently amplified using GeneAmp RNA PCR Kit (Life Technologies Ltd, Paisley, UK). The primers used for TMPAP amplification by RT-PCR were: 5′-AATCTAGACCATGCCAGCCGTTCCT-3′ (forward) and 5′- CTCTCTAGATCAGATTGTTCCGATACAC-3′ (reverse). The PCR conditions were: 95°C for 1 min and 45 s, followed by 30 cycles of 95°C for 15 s, 63.4°C for 30 s, and 72°C for 1 min and 12 s, with the final extension of 7 min at 72°C. PCR product was cloned into pCR2.1 TOPO vector (Life Technologies Ltd.), and bidirectionally sequenced.

### Electrophysiology

Hippocampal slices (350 µm) were cut with a vibratome from postnatal day (P) 14–18 WT or PAP*^−/−^* mice (n = 14–16) using standard methods [18]. The slices were used 1–4 h after cutting. For electrophysiological recordings, the slices were placed in a submerged chamber and superfused with artificial cerebrospinal fluid (ACSF) containing: 124 mM NaCl, 3 mM KCl, 1.25 mM NaH_2_PO_4_, 1 mM MgSO_4_, 26 mM NaHCO_3_, 15 mM d-glucose and 2 mM CaCl_2_; 5% CO_2_–95% O_2_, at a rate of 2–3 ml/min (32°C). Whole-cell recordings were obtained from CA1 pyramidal neurons by using the Multiclamp 700B amplifier (Molecular Devices, Sunnyvale, CA, USA). Cells were voltage-clamped at 0 mV with 4–5 MΩ pipettes filled with a solution containing: 135 mM CsMeSO_4_, 10 mM Hepes, 0.5 mM EGTA, 4 mM Mg-ATP, 0.3 mM Na-GTP, 5 mM N-(2,6-dimethylphenylcarbamoylmethyl) triethylammonium chloride and 2 mM NaCl (285 mOsm), pH 7.2. GABAA-mediated mIPSCs were recorded in the presence of 1 µM TTX, 10 µM 2.3-dihydroxy-6-nitro-7-sulfamoylbenzo(f)quinoxaline (NBQX), 1 µM 3-N[1-(S)-(3,4-dichlorophenyl)ethyl,] amino-2-(S)-hydroxypropyl-P-benzyl-phosphinic acid (CGP55845) and 50 µM D-2-amino-5-phosphonovalerate (D-AP5). All compounds were from Tocris (Bristol, UK). Axoscope 10.2 (Molecular Devices) was used for data acquisition. Offline analysis was done using MiniAnalysis 6.0.7 program (Synaptosoft, GA, USA). Spontaneous events were detected using peak detector algorithm, and all events were confirmed visually. Rise and decay times were measured between 20 and 80% and 90–37% of the peak amplitude, respectively. The histograms and cumulative distributions were constructed from at least 10 min of recording (at least 50 events) from each cell, using a bin width of 100 ms for inter-event interval and 1 pA for amplitude. The pooled data are given as mean ± S.E.M. for the number of cells indicated.

## Results

**Figure 1:**
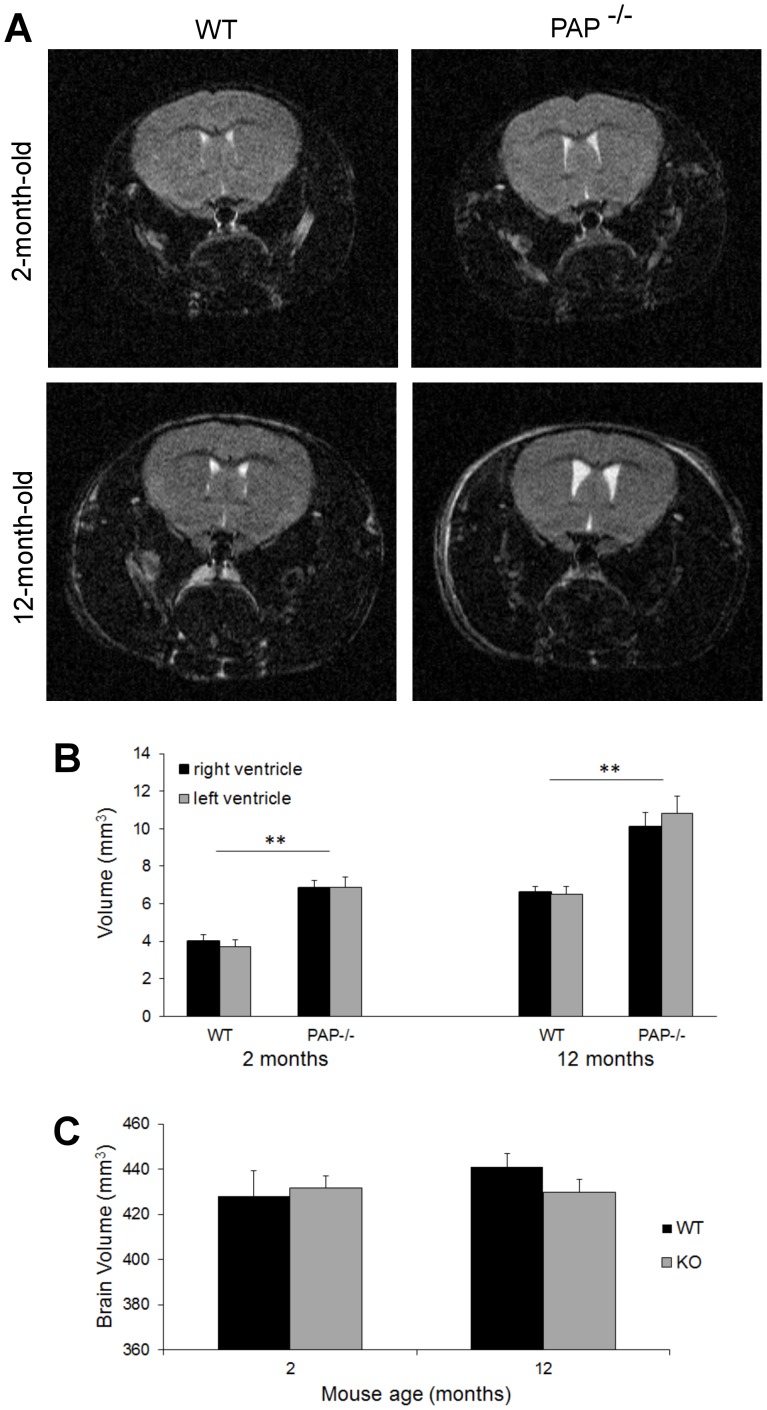
Lateral ventricles volume is enlarged in PAP^−/−^ mice. Lateral ventricles (right and left ventricles) volume is significantly larger (**p<0.01) in both young and old PAP*^−/−^* mice compared to corresponding WT mice. (A) T2-weighted images from young (2 months) and old (12 months) WT and PAP*^−/−^* mice. Plot of (B) lateral ventricle volumes and (C) total brain size for WT and PAP*^−/−^* mice. The data is expressed as mean±S.E.M.

**Figure 2:**
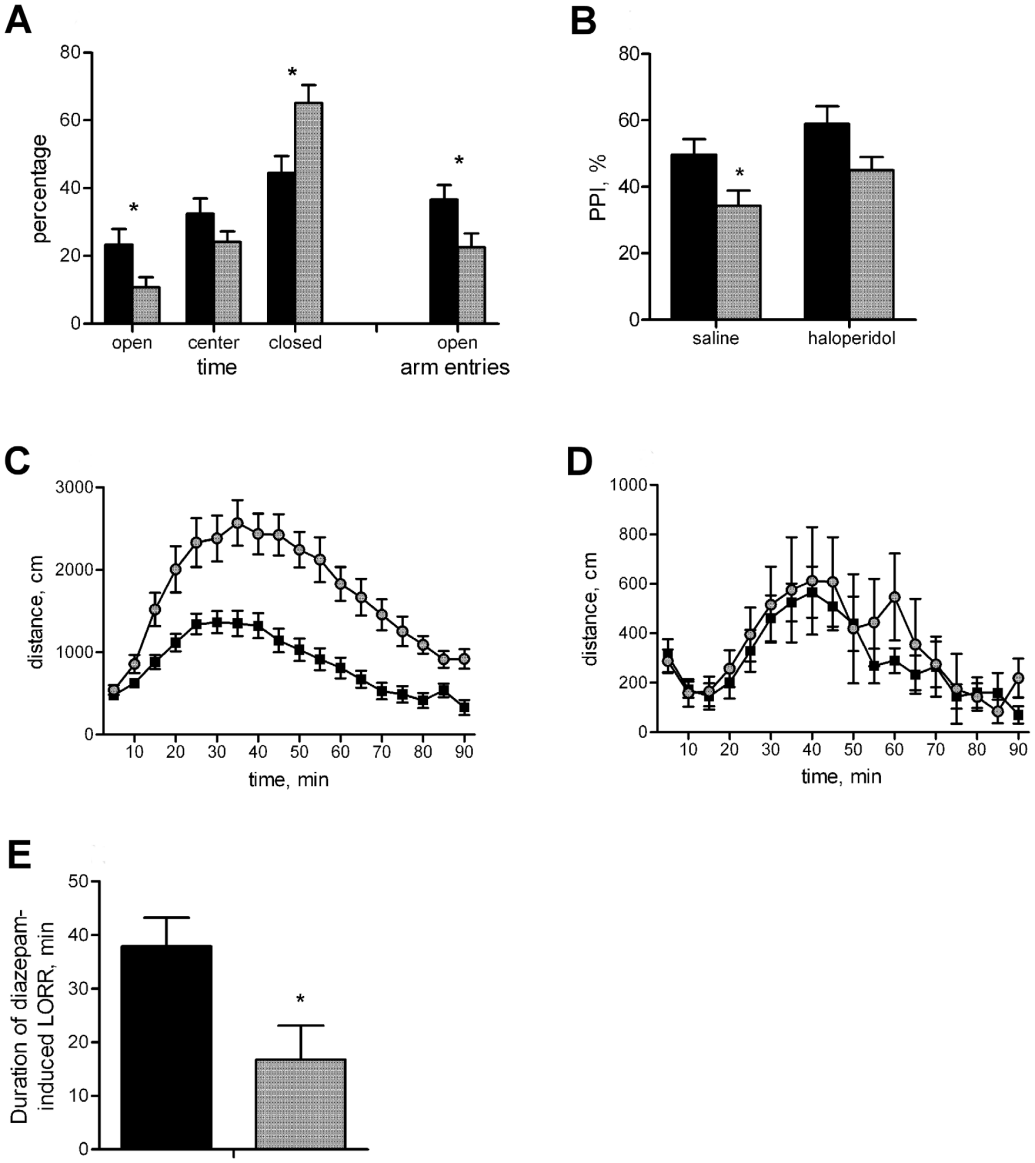
PAP^−/−^ mice show increased anxiety, disrupted PPI, augmented locomotor response to amphetamine and increased tolerance to diazepam-induced loss of righting reflex. (A) PAP^−/−^ mice spent less time in open arms of the elevated plus maze, and more time in closed arms than WT mice. The ratio of open arm entries was lower in PAP^−/−^ mice. (B) PAP^−/−^ mice have reduced PPI of the acoustic startle reflex. PPI was enhanced by treatment with haloperidol. (C) Amphetamine-induced locomotor activity was significantly increased in PAP^−/−^ mice compared to WT mice (p = 0.0001), whereas the effect of MK-801 (D) was similar between the genotypes. (E) PAP^−/−^ mice displayed shorter duration of loss of righting reflex after treatment with high dose of diazepam. The data is expressed as mean±S.E.M.; black bars/squares represent WT, grey bars/dots PAP^−/−^ mice; *p<0.05 between the genotypes.

To assess the function of TMPAP in the CNS, we first evaluated the anatomy of the brain and performed MRI analyses of WT and PAP^−/−^ mice. The results revealed that PAP*^−/−^* mice had significantly enlarged lateral brain ventricles (Fig. 1A and B) when compared to WT. However, there was no significant difference in brain size between genotypes (*p*>0.05, Fig. 1C), and the brain sizes obtained were in agreement with published information [19]. Since PAP*^−/−^* mice had significantly enlarged lateral brain ventricles we subjected them at first to a detailed behavioral analysis (Table 1). PAP*^−/−^* mice displayed increased anxiety in the elevated plus-maze test (Fig. 2A) and had a disruption in prepulse inhibition (PPI), which indicates a defect in the sensorimotor gating system. The administration of the typical antipsychotic drug haloperidol improves the response of PAP^−/−^ mice in the PPI test (Fig. 2B). In PPI of the startle reflex: initial screening showed significantly reduced PPI in KO mice [F(1,16) = 9.7, p = 0.0067), therefore we tested the effect of haloperidol (0.2 mg/kg) on PPI. Factorial ANOVA with genotype (WT, KO) and treatment (saline, haloperidol) as independent variables revealed significant main effect of genotype [F(1,55) = 9.9, p = 0.0027] and treatment [F(1,55) = 4.6, p = 0.0368]. There was no interaction between the genotype and treatment. However, post-hoc Newman-Keuls test revealed that treatment with haloperidol increased the PPI of KO mice to the level of WT mice treated with saline (p = 0.48) and no difference was observed between KO and WT mice treated with haloperidol (p = 0.09). Significant difference between saline treated KO and WT mice was still evident (p = 0.05). PAP*^−/−^* mice also showed an augmented response to the psychostimulant D-amphetamine (Fig. 2C). However, the locomotor response of PAP*^−/−^* mice to MK-801, a NMDA receptor blocker and a compound used to mimic psychosis in rodents, was normal (Fig. 2D), suggesting that PAP*^−/−^* mice have changes in their dopaminergic system. PAP*^−/−^* mice were also less sensitive to diazepam-induced loss of righting reflex (Fig. 2E), indicating alterations in GABAergic functions. PAP*^−/−^* mice showed no defects in tests measuring learning, memory, motor coordination, social interaction or depression-like behavior (Table 1).

**Table 1:** Anatomical, neurochemical and behavioral characterization of mice deficient in prostatic acid phosphatase (PAP).

Characteristic	PAP^−/−^ mice
Enlarged lateral ventricles	Yes
Dopamine	↑ DA synthesis ↑ DOPAC ↑ DOPAC/DA
Gamma-aminobutyric acid (GABA)	↑ frequency of GABAA-mediated mIPSC
Haloperidol response	Yes
Diazepam-induced LORR	Reduced
Sensorimotor gaiting (PPI)	Reduced
Amphetamine hypersensitivity	Increased
MK-801 hypersensitivity	NSD
Anxiety-like behavior (EPM)	Increased
Anxiety-like behavior (LD)	NSD
Novelty-induced activity (OF)	NSD
Short-term memory (Y-maze)	NSD
Motor coordination (RR)	NSD
Motor coordination (BW)	NSD
Depression-like behavior (FST)	NSD
Depression-like behavior (TST)	NSD
Associative learning (FC)	NSD
Social dominance (Tube-test)	NSD
Social interaction (RI)	NSD
Spatial learning and memory (WM)	NSD

**ABBREVIATIONS:** NSD - no significant difference; ↑- increased; DA - dopamine; DOPAC - 3,4-Dihydroxyphenylacetic acid; GABAA - GABAA receptor; mIPSC - miniature inhibitory postsynaptic current; LORR - loss of righting reflex; PPI - prepulse inhibition; EPM - elevated plus-maze; OF - open field; LD - light-dark box; RR - rota-rod; BW - beam walking; FST - forced swim test; TST - tail suspension test; FC - fear conditioning; RI - resident-intruder test; WM - water maze.

**Figure 3:**
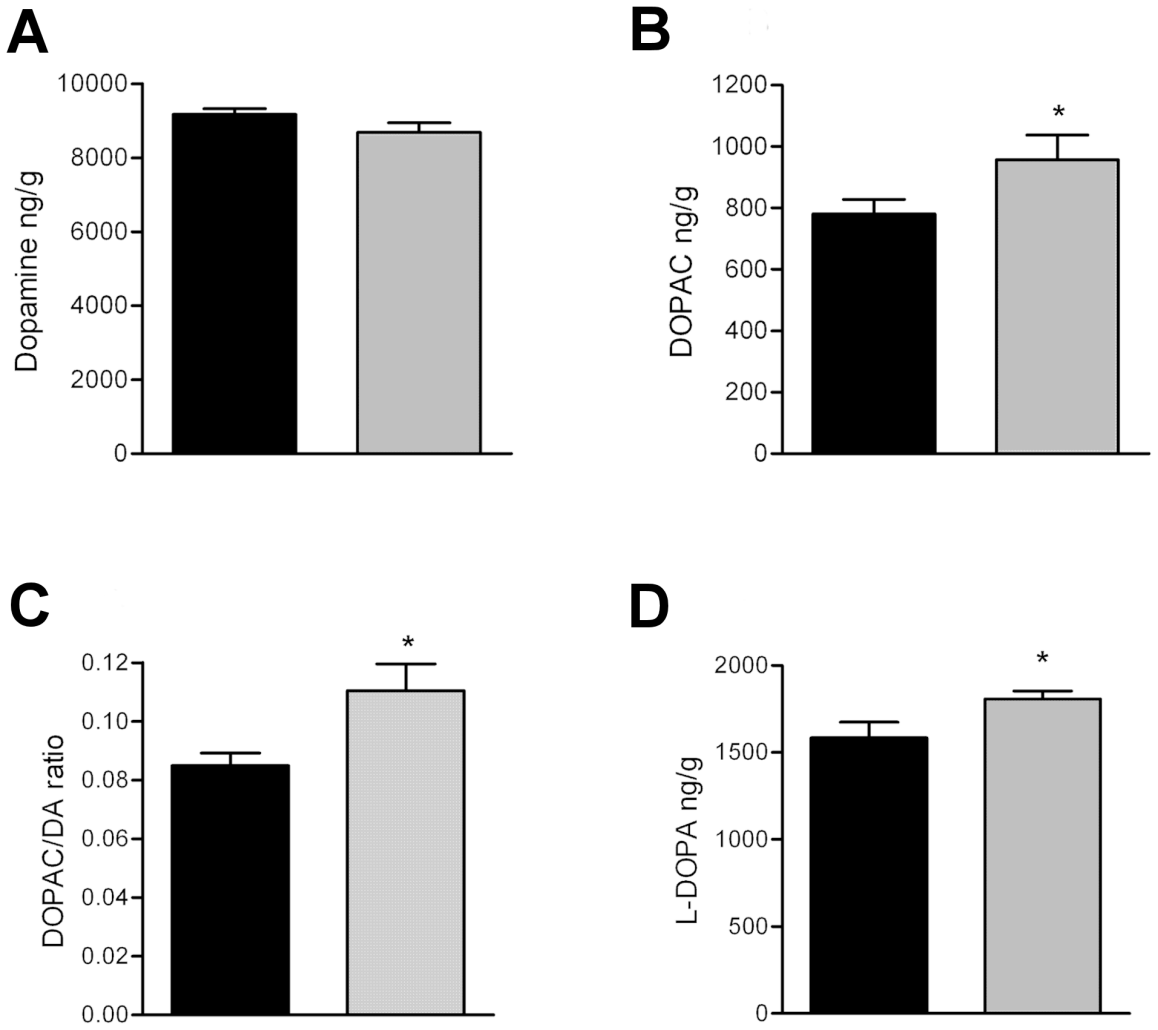
Dopamine synthesis is augmented in the striatum of PAP*^−/−^* mice. (A) Tissue levels of DA are similar in WT and PAP*^−/−^* mice. (B) Level of the principal metabolite of DA, DOPAC, is elevated in the PAP*^−/−^* mice, and also the DOPAC/DA ratio is elevated (C). (D) Accumulation of L-DOPA is greater in PAP^−/−^ mice than WT mice 30 min after administration of a blocker of L-amino acid decarboxylase, indicating increased DA synthesis. The data is expressed as mean±S.E.M.; black bars represent WT, grey bars PAP^−/−^ mice; *p<0.05, two tailed t-test (monoamines) or repeated measures ANOVA (microdialysis).

Due to the plausible changes in the dopaminergic system of PAP^−/−^ mice, we measured the levels of dopamine (DA) and its metabolites from the striatum of PAP*^−/−^* mice by microanalysis, and no notable difference were observed in the tissue concentration of DA (Fig. 3A). However, the level of its primary metabolite, DOPAC, was increased 20% (Fig. 3B). The DOPAC/DA ratio was also increased 30%, indicating increased synthesis or turnover of DA (Fig. 3C). To discern which mechanism was causing the increment in DOPAC, we studied DA synthesis rate by blocking the dopadecarboxylase enzyme which converts L-dopa to DA by using NSD1015 as inhibitor, and measured the accumulation of L-dopa as an index of DA synthesis [20], and observed that the accumulation of L-DOPA was 14% higher in PAP*^−/−^* mice, verifying augmented synthesis of DA (Fig. 3D).

**Figure 4:**
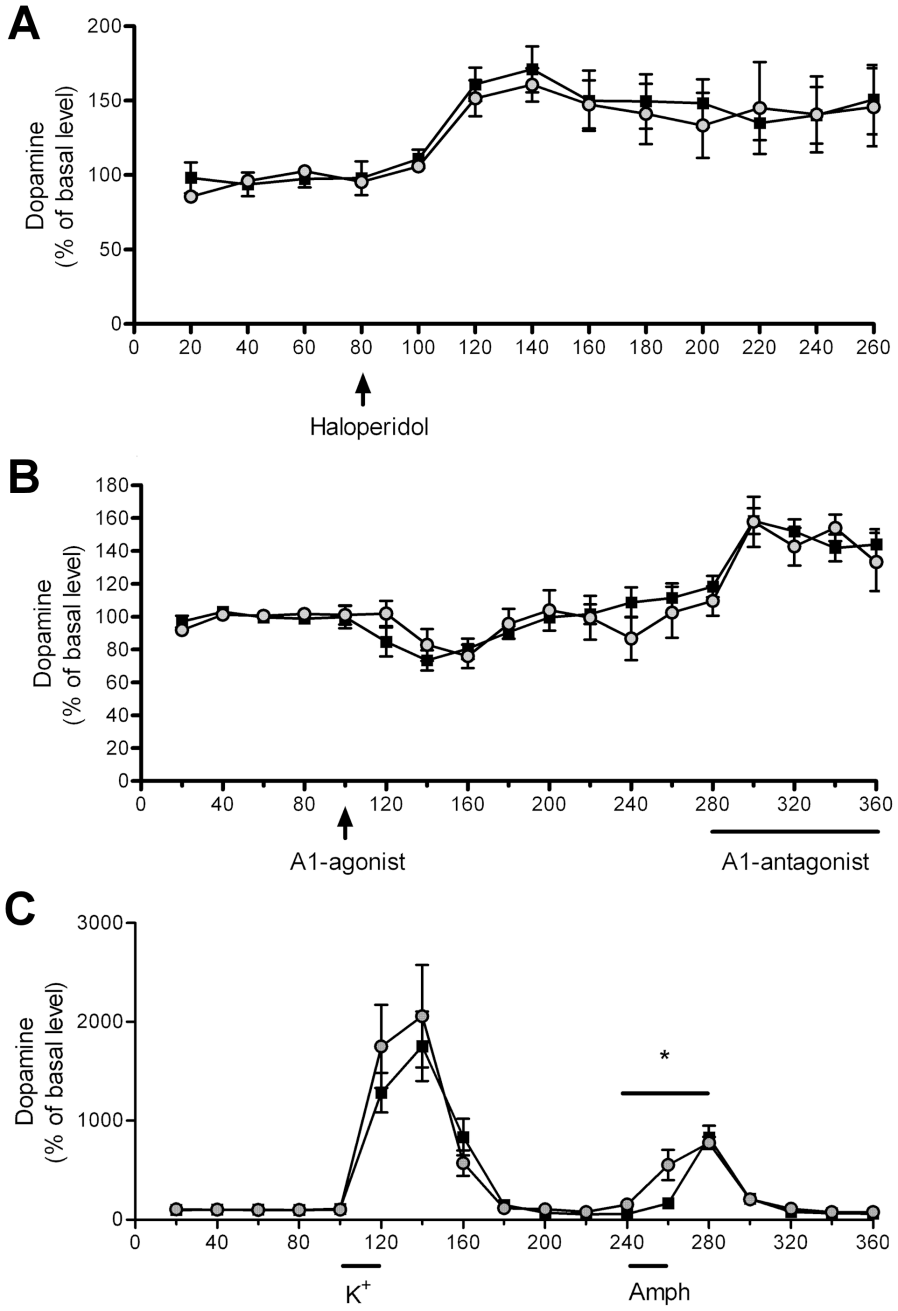
Characterization of dopaminergic transmission in PAP^−/−^ mice by microdialysis. (A) There is no difference in haloperidol (0.2 mg/kg) -induced dopamine release between PAP^−/−^ and WT mice. (B) Adenosine A_1_-agonist GR79236X (1 mg/kg) and A_1_-antagonist 8-CPT (300 µM in dialysis fluid) significantly decreased and increased the release of dopamine, respectively (repeated measures ANOVA) but there was no difference in the magnitude of response between the genotypes. (C) There is no significant difference in potassium–induced DA release between the genotypes, but amphetamine induces DA release significantly faster. The data is expressed as mean±S.E.M.; black squares represent WT, grey dots PAP*^−/−^* mice.

To further characterize the dopaminergic transmission in *PAP^−/−^* mice we conducted a series of microdialysis experiments. Given that DA D_2_-autoreceptors control the synthesis of dopamine, we tested the difference in D_2_-receptor function in PAP*^−/−^* mice by administering haloperidol, but observed no significant difference between the genotypes indicating normal D_2_-receptor function in PAP*^−/−^* mice (Fig. 4A). As we previously showed that PAP produces adenosine that alleviates pain via A_1_ receptors [9], and A_1_ receptors modulate DA release [21],we also tested striatal dopaminergic response to adenosine A_1_-receptor agonist or antagonist in PAP*^−/−^* mice by microdialysis, but saw no difference between the genotypes, suggesting no difference in the sensitivity of A_1_ receptors (Fig. 4B). Next, we tested whether there is a difference between both genotypes in the capacity to release DA by stimulating DA release with high concentration of potassium or D-amphetamine in the dialysis fluid. We found that D-amphetamine induces DA release significantly faster in the PAP^−/−^ than in the WT mice (Fig. 4C).

**Figure 5:**
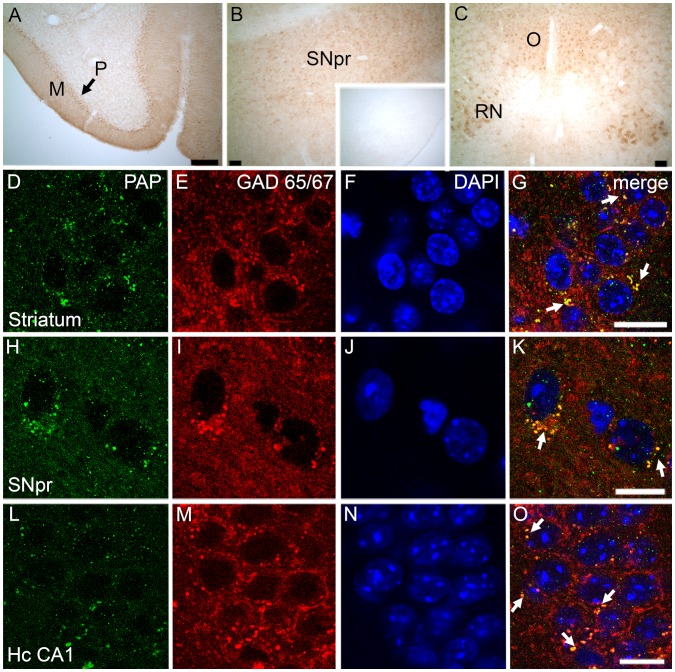
TMPAP is expressed in the mouse brain and colocalizes with GABAergic marker, GAD 65/67. Representative confocal images depict intense TMPAP (brown color) expression in molecular cell layer (M) and Purkinje cells (P) of cerebellum (Panel A), in substantia nigra pars reticulata (SNpr; Panel B), in red nucleus (RN; Panel C) and in oculomotor nucleus (O; Panel C). Small picture in Panel B depicts the TMPAP staining of the substantia nigra in PAP*^−/−^* mouse. TMPAP (green) was colocalized with GABAergic marker (red) in medium spiny neurons of striatum (Panels D-G, yellow color and white arrows indicating the colocalization) and in SNpr (Panels H–K). Colocalization was evident also in GABAergic neurons of hippocampus CA1 (Panels L–O). DAPI (blue color) was used as a nuclear marker. Scale bars are 500 µm in Panels A–C, and 10 µm in panels D–O.

These anatomical, neurochemical, and behavioral characteristics observed in PAP*^−/−^* mice suggested that PAP has an important role in the central nervous system and prompted us to characterize its localization and expression in the mouse brain. Immunohistochemical stainings showed that TMPAP is widely expressed in motor-related brain areas, with the most intense PAP-immunoreactivity in cerebellar Purkinje cells, red nucleus, oculomotor nucleus, and in substantia nigra pars reticulata (Fig. 5A–C), and no PAP-immunoreactivity was seen in PAP*^−/−^* sections (Fig. 5 B inset). We also cloned a full-length TMPAP transcript from mouse striatal neurons (see Materials and Methods), verifying expression of TMPAP. No sPAP transcript was detected.

We queried the Allen Brain Atlas database [22] for expression of PAP in mouse and human brain. *In situ* hybridization data of the mouse brain was only available for sPAP (NM_019807), and showed no expression of sPAP in the mouse brain, consistent with our cloning findings. Gene expression data from the developing human brain showed high levels of PAP expression (both sPAP and TMPAP isoforms) in the ventricular and subventricular zones [22].

**Figure 6:**
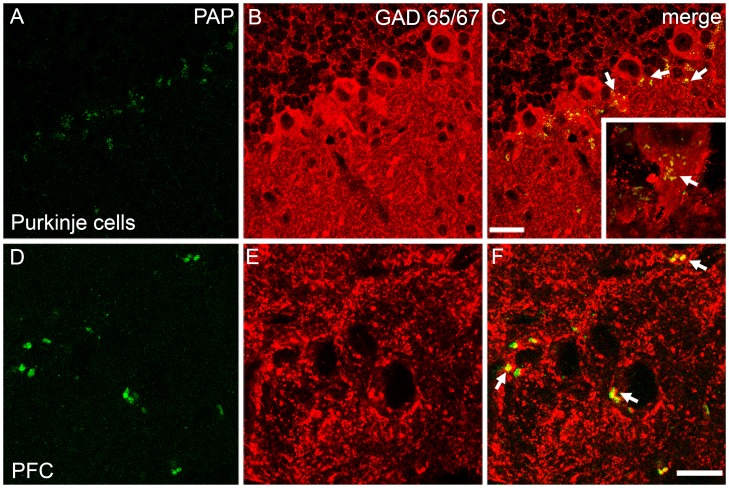
The colocalization of PAP (green) and GAD65/67 (red) was seen in several areas of brain. In cerebral Purkinje cells (A–C), strong colocalization was seen especially in the axon hillock of the neuron (small picture in C; yellow color and white arrows depicting the colocalization). Similarly, PAP was present in GABAergic neurons in prefrontal cortex (PFC; infralimbic cortex) (D–F). Scale bars are 10 µm.

Immunostaining revealed clear colocalization of TMPAP with GABA-specific marker GAD65/67 in several brain areas (Fig. 5D–O) (Fig. 6A–F), but hardly any TMPAP immunoreactivity outside of GABAergic neurons. TMPAP was preferentially located in the axon hillocks of GABAergic neurons (Fig. 6A–C) where GABAergic synapses are located [23].

**Figure 7:**
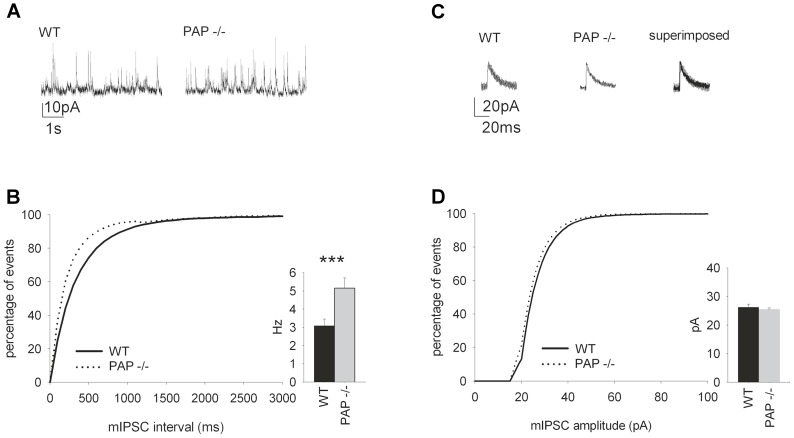
The frequency of spontaneous hippocampal mIPSCs is increased in PAP^−/−^ mice. (A) Sample traces of mIPSC from WT and PAP*^−/−^* mice (P14–P18). (B) The mIPSC frequency in the PAP*^−/−^* mice was higher (*p* = 0.003) compared to WT mice. The graph shows the cumulative distribution of the events. (C) Sample traces from 8 averaged mIPSCs from PAP*^−/−^* and WT mice. (D) There were no differences in the amplitude in mIPSCs recorded from WT and PAP*^−/−^* mice. The graph shows the cumulative distribution of the amplitudes.

We next performed whole-cell patch-clamp recordings from hippocampal CA1 pyramidal cells and observed increased frequency of GABAA receptor-mediated mIPSCs in PAP*^−/−^* mice (Fig. 7A–B). There was no change in the mIPSC amplitude (Fig. 7C–D).

**Figure 8:**
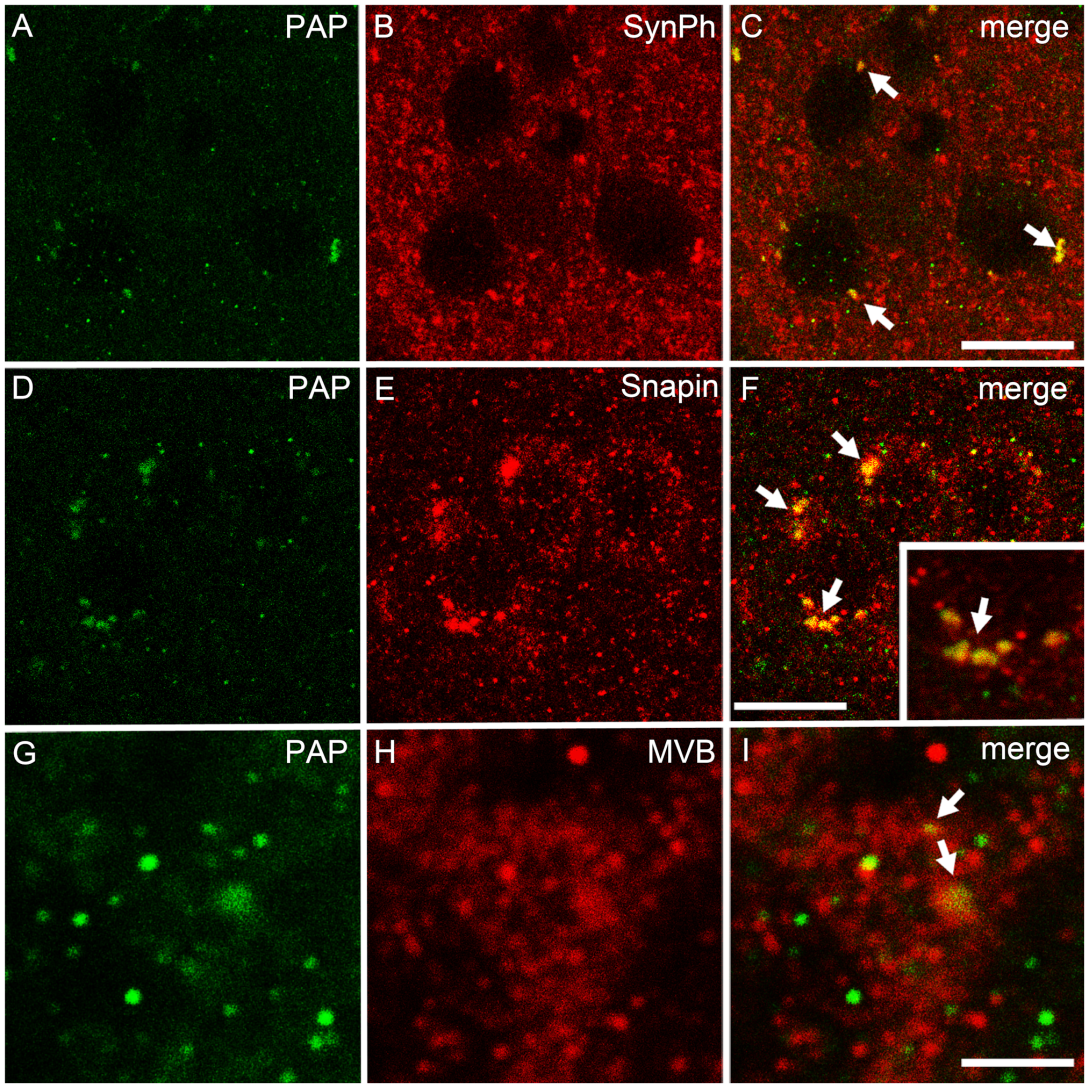
Immunofluorescent colocalization stainings of TMPAP and synaptic vesicle associated proteins. TMPAP (green) is colocalized with a presynaptic marker, synaptophysin (red) (A–C; yellow color and white arrows depicting the colocalization). PAP was seen in vesicle-like structures that had strong colocalization with Snapin (D–F). Small picture is a magnification from panel C, depicting the colocalization. Moreover, largest PAP-immunoreactive structures had a colocalization with multivesicular bodies (MVB, red; G–I). All pictures are from striatum. Scale bars are 10 µm in A–C and G–I, and 3 µm in D–F.

**Figure 9:**
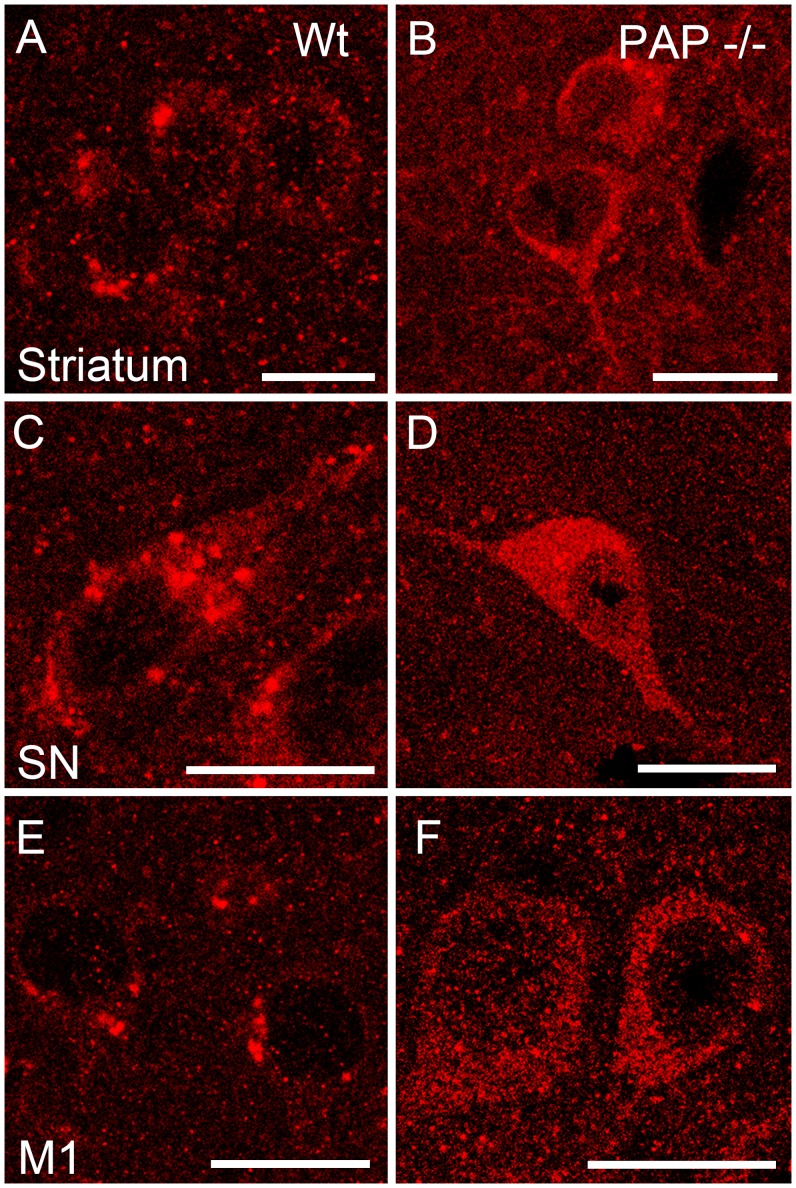
Immunostaining of Snapin in various brain areas of WT and PAP^−/−^ mice. In the PAP^−/−^ mouse, Snapin (red) is localized more diffusely in the cell soma (B, D, F). Cellular localization of Snapin in WT mouse is more vesicular-like (A, C, E). Scale bar is 10 µm in all figures. M1 - primary motor cortex; SN - substantia nigra.

We have previously shown that TMPAP is localized presynaptically in the dorsal root ganglia and spinal cord [9]. Therefore, we performed further colocalization studies of TMPAP with synaptophysin, a marker of presynaptic nerve endings [24], and found colocalization in large, vesicle-like structures (Fig. 8A–C), suggesting that TMPAP is located at the presynaptic compartment also in the brain. Since TMPAP colocalized with bis(monoacylglycero)phosphate, a marker for multivesicular bodies [1], and interacts with snapin [3], we carried out double-immunostaining of TMPAP and snapin, as well as TMPAP and anti-CHMP2B, also a marker for multivesicular bodies. The immunofluorescence stainings revealed colocalization of these proteins (Fig. 8D–I), which suggests that cerebral TMPAP is also involved in endo−/exocytosis. The lack of TMPAP in our PAP^−/−^ model also produces a differential localization of snapin in the cells. In WT animals snapin resides in vesicular structures, whereas in PAP*^−/−^* mice snapin is localized more diffusely in the cell soma (Fig. 9).

## Discussion

The anatomical, behavioral and neurochemical changes observed in PAP*^−/−^* mice (summarized in Table 1) suggest that PAP has important supraspinal functions. The enlargement of lateral ventricles has been observed in human neurodegenerative diseases such as Alzheimer’s disease, dementia, bipolar disorder and schizophrenia; and also in movement disorders like Parkinson’s and Huntington’s diseases [25]–[29]. A query of the Allen Brain Atlas shows that in the adult human brain, PAP is highly expressed in areas related to language, motor coordination, cognitive function, and self-awareness; and PAP is also highly expressed in the ventricular and subventricular zones of the developing human brain [22]. We have not addressed the expression of PAP in the developing mouse brain, but if like in humans PAP is expressed during development in the ventricular and subventricular zones, this could affect the development of the brain and be an explanation for the enlarged lateral ventricles observed in the PAP^−/−^ mice. It is important to mention that previous studies have shown an increased incidence of spontaneous hydrocephaly in C57BL/6 mice compared to other mouse strains [30]. Dahme and coworkers have shown that mutation in the adhesion molecule L1 gene produces enlarged lateral ventricles in mice with C57BL/6J background but this change was absent in mice with 129/SvEv background [31]. Hence, the observed phenotype of enlarged lateral ventricles in PAP^−/−^ mice might be partially dependent on genetic background. However, enlarged ventricles were only observed in PAP^−/−^ mice compared to WT mice. Additionally, it has been shown that GABAergic neurons regulate lateral ventricular development [32].

The phenotype of PAP*^−/−^* mice is most likely not related to adenosine, since there was no difference in striatal dopaminergic response to adenosine A_1_-receptor agonist or antagonist, suggesting that other adenosine-synthesizing enzymes expressed in the brain [33]–[35] most likely compensate for the lack of TMPAP. The effect of amphetamine on locomotor activity of PAP*^−/−^* mice was augmented, which suggest altered dopaminergic response either in the level of dopamine receptors or in the indirect mechanisms controlling dopamine release. Indeed, microdialysis studies showed that effect of amphetamine on striatal dopamine release was more rapid in the PAP*^−/−^* mice. Since the haloperidol-induced dopamine release was not altered in the PAP*^−/−^* mice, their direct D2-receptor mediated control of DAergic transmission seems to be normal. Thus, indirect mechanisms are more likely to be involved. As amphetamine has a depressant effect on the firing rate of DAergic neurons through the striatonigral neuronal feedback loop [36], it is possible that the striatonigral GABAergic feedback pathway controlling DA release is not functioning normally, resulting in augmented response to amphetamine.

In addition, our results of whole-cell patch-clamp recordings from hippocampal CA1 pyramidal cells show the increased frequency of mIPSCs in PAP*^−/−^* mice, suggesting either an increase in the GABA release or in the density of GABAergic synapses. The increase in GABAergic tone in PAP*^−/−^* mice is also in line with their decreased sensitivity to diazepam, as in the presence of increased GABAergic tone the allosteric upmodulation of GABAA receptors is likely to be less effective. On a cellular level, how could TMPAP regulate GABAergic transmission to produce such a distinct phenotype? TMPAP has an endosomal/lysosomal targeting signal, and in prostate cancer tissue it is localized in multivesicular endosomes and lysosomes, as well as luminal exosomes [1], [3]. In this study we have shown by colocalization with CHMP2B that also in neurons TMPAP is in multivesicular endosomes. In nerve cells, these organelles are used in membrane trafficking pathways controlling recycling and degradation of pre- and post-synaptic membrane proteins, as well as in recycling of vesicle membrane during neurotransmitter release [37] and release of exosomal endocargo [38].

The results of the colocalization studies with synapthophysin and snapin, suggest that TMPAP is localized in synaptic nerve endings. This conclusion is in agreement with previous studies which show that mouse PAP localized presynaptically in DRG neurons [9] and in taste buds [39]. In addition, the colocalization between PAP and snapin in prostate cancer cells occurs in the cell lamellipodia [3], and it has been described that the lamellipodium is the site where exocytosis occurs in migrating mammalian cells [40], [41], supporting the hypothesis that colocalization of these proteins in the neuron will not happen in the cell soma. This presynaptic localization of TMPAP together with the fact that TMPAP resides in the axon hillock, where GABAergic synapses are located [23], supports the hypothesis that TMPAP is located in GABAergic synapses. TMPAP also colocalizes and interacts with snapin [3], which directly binds SNAP-25 [42], a protein that has been linked to schizophrenia in genetic [43], pathological [44]–[46] and functional studies [47]–[49]. Snapin is associated with the SNARE complex and involved in synaptic vesicle docking and fusion, supporting the hypothesis that TMPAP may regulate GABAergic signaling via synaptic vesicle trafficking. The mislocalization of snapin observed in the cells of PAP*^−/−^* mice may perturb synaptic processes controlling neurotransmitter release and recycling, thus disrupting neuronal homeostasis and eventually leading to the neurological phenotype observed in PAP*^−/−^* mice.

Enlarged lateral ventricles are present in numerous neurological disorders such as schizophrenia, Alzheimer’s disease, bipolar disorders, Parkinson’s disease and Huntington’s disease as well as in many mouse models of the diseases [50]–[53]. Also, decreased prepulse inhibition (PPI) is considered a behavioral endophenotype of schizophrenia [54]. To our knowledge, mutations in the gene encoding PAP (*ACPP*) have not thus far been reported, nor has *ACPP* been implicated in genetic association studies of mental disorders. However, the SNAP-25 locus (Chr: 20p12.3-11) has been implicated in a meta-analysis of genome-wide linkage scans of schizophrenia [43]. GABAergic dysfunctions and alterations in inhibitory circuits of the brain have been implicated in several mouse models of schizophrenia and related disorders [55]). We suggest that by influencing GABAergic signaling through vesicle trafficking, TMPAP might be linked to specific endophenotypes seen in neurological and neuropsychiatric disorders.
